# Influence of maternal or neonatal Vitamin A injection on calf performance and muscle transcriptomics in the cow-calf phase

**DOI:** 10.1007/s11250-026-04976-0

**Published:** 2026-03-14

**Authors:** J. M. Oliveira, D. R. Casagrande, S. A. S. Laura, A. H. A. Horta, A. S. Miranda, T. R. S. Gionbelli, L. H. L. Chalfun, M. M. Ladeira

**Affiliations:** 1 Department of Animal Science, Universidade Federal de Lavras, Lavras, Minas Gerais 37200-900 Brazil; 2 Centro Universitário de Lavras - Unilavras, Lavras, Minas Gerais 37203-593 Brazil

**Keywords:** Adipogenesis, Fetal programming, Muscle growth, Myogenesis, Neonatal programming, Retinoic acid

## Abstract

This study investigated the effects of vitamin A (VA) injection on growth performance, muscle development, and skeletal muscle transcriptomics in calves during the cow-calf phase. Pregnant cows at 250 days of gestation were randomly assigned to one of three treatments: control (CON; no VA injection), VA injection in cows at 250 d of gestation (VAcow), or VA injections in calves at birth and 60 days of age (VAcalf). Calves were weighed at 60 d and weaning. Carcass ultrasound and muscle biopsy were conducted at weaning. Gene expressions were analyzed by RT-qPCR. Male calves in the VAcow group had higher (*P* = 0.01) ADG than Control males and VA-treated females. Weaning BW was greater (*P* < 0.01) in VAcalf and VAcow males compared to Control males and VA-treated females. Gene expression analysis revealed that *RARA*, *MYF5*, *CEBPA*, and *DLK1* were higher (*P* ≤ 0.05) expressed in Control and VAcow calves than in VAcalf calves. Expressions of *ZNF423* and *RXRA* tended to be greater (*P* ≤ 0.09) in VAcow males than in VAcalf males and VA-treated females. The expression of *PAX7* was higher (*P* < 0.01) in VAcow males than in all other groups. Also, *IGFR1* expression tended to be greater (*P* = 0.07) in VAcow males than in Control and VAcalf males and VAcalf females. In conclusion, VA injection during late gestation or early postnatal life improved growth of male calves. Maternal VA injection had a more pronounced effect on muscle gene expression, particularly in male calves.

## Introduction

Vitamin A is a fat-soluble substance that plays important roles in numerous biological processes. Its bioactive functions in mammalian physiology and metabolism have been recognized for decades, especially in key pathways that influence animal growth, health, and productivity. In recent years, Vitamin A has also emerged as a potential regulator of fetal and early postnatal development, particularly in muscle and adipose tissues (Ladeira and Oliveira Júnior, 2022). According to Du et al. (2010), the last trimester of gestation and early neonatal period are the critical window to adipogenesis in beef cattle. During these stages, mesenchymal progenitor cells in muscle tissue differentiate into adipocytes, which later accumulate lipids to form intramuscular fat. The abundance and differentiation potential of these progenitor cells decline as the animal ages, making early intervention most effective for enhancing marbling. The role of Vitamin A in intramuscular fat (IMF) deposition and muscle growth has gained increasing attention, especially when administered during late gestation or early postnatal life in cattle (Wang et al. 2018; Harris et al. 2018; Jo et al. 2020; Maciel et al. 2022; Peng et al. 2020; Dean et al. 2024).

Mechanistically, VA regulates key molecular pathways that drive adipogenesis and angiogenesis. Yu et al. (2022) demonstrated that VA upregulates *Vascular Endothelial Growth Factor (VEGF)* expression, stimulating the development of intramuscular capillaries that serve as a niche for adipose progenitor. In addition, the active form of Vitamin A, retinoic acid (RA*)*, forms a complex with Retinoid X Receptor (RXR) and Retinoic Acid Receptor (RAR), which then binds to DNA and alters transcription of target genes (Chawla et al. 2001; Rochette-Egly and Germain 2009). Finally, another adipogenic transcription factor, the Peroxisome Proliferator-Activated Receptor Gamma (PPARG), requires a heterodimer formation with RXR to regulate mRNA synthesis.

Several studies have explored Vitamin A administration in cattle through different routes and developmental stages. Harris et al. (2018) showed that two postnatal injections of Vitamin A (at birth and 1 month of age; with 150,000 or 300,000 IU) increased IMF at weaning and slaughter, with the 150,000 IU dose being most effective. Aiming to simplify management, Maciel et al. (2022) demonstrated that a single Vitamin A injection at birth (300,000 IU) also enhanced IMF deposition. Additionally, maternal or oral supplementation during pregnancy has shown positive effects on offspring development. Jo et al. (2020) reported that supplementing pregnant cows with 78,000 IU/d from 225 d of gestation to delivery increased calf birth weight and upregulated genes related to muscle and preadipocyte development. Similarly, Dean et al. (2024) observed that feeding pregnant cows higher dietary Vitamin A levels (12.2 KIU/kg) from day 180 of gestation to parturition improved IMF content and enhanced expression of key genes and proteins associated with adipogenesis.

Therefore, the cited studies indicate that Vitamin A can modulate fetal and early postnatal development, influencing both muscle and adipose tissue programming. However, to date, no published study has evaluated the effects of Vitamin A injection in pregnant cows. This represents a critical knowledge gap, since the injection may provide more precise and consistent delivery of Vitamin A compared to oral sources, overcoming variation in intake and absorption during gestation. Exploring the effects of maternal Vitamin A injection may therefore reveal another practical and efficient strategy for developmental programming in beef cattle.

Beyond its role in the IMF, Vitamin A supplementation has also been associated to improved growth performance. In addition to enhanced IMF deposition, Harris et al. (2018) observed greater average daily gain (ADG) and consequently heavier BW at weaning (210 d) and at 308 d of age in Vitamin A-treated calves. These findings prompted further investigation into VA’s influence on muscle fiber formation and metabolism. Wang et al. (2018) reported that Vitamin A upregulated myogenic transcription factors, increased protein expression, satellite cells density, muscle fiber cross-sectional area, and oxidative fiber proportion. These findings align with those of Jo et al. (2020) and Peng et al. (2020), who also reported upregulation in myogenic factors following Vitamin A treatment.

The timing of nutritional intervention is crucial for programming muscle and adipose tissue development. Adipogenesis of intramuscular adipose tissue peaks in late pregnancy and extends until 250 d of age, a period known as the marbling window (Du et al. 2015). In contrast, myogenesis predominantly occurs during the prenatal phase, with primary myofibers forming during the first trimester and secondary myofibers during the second trimester of gestation (Du et al. 2010). Final fiber maturation, including contractile and metabolic differentiation, occurs in late gestation (Du et al. 2010; Picard et al. 2002). Therefore, late pregnancy represents a critical windown which maternal Vitamin A administration could simultaneously influence fetal myogenic and adipogenic pathway, enhancing traits of economic importance for beef production.

So we hypothesized that Vitamin A injection in pregnant cows would elicit developmental programming effects similar to or greater than those observed in calves receiving Vitamin A postnatally, enhancing offpring growth performance, myogenesis, angiogenesis, and adipogenesis. Thus, the objectives of the present study were to evaluate the effects of Vitamin A injection on growth performance, muscle development, and skeletal muscle transcriptomics in Angus × Nellore animals during the cow-calf phase.

## Materials and methods

All experimental procedures and protocols in this study were approved by the Ethics Committee on Animal Use of the Universidade Federal de Lavras (UFLA; protocol 014/21). The trial was carried out at a commercial farm (MS Pecuária) located in Extrema, Minas Gerais, Brazil. The analyses were performed in the Molecular Biology Laboratory of the Department of Animal Science at UFLA (Lavras, Minas Gerais, Brazil).

### Experimental design, treatments, and animals

Originally, a total of 51 Nellore cows (initial BW of 438 ± 48.9 kg; initial body condition score (BCS) of 5.1 ± 0.97) were utilized in a randomized complete block design with a 3 × 2 factorial arrangement (3 treatments and 2 offspring sexes). Cows were weighed, blocked by parity, and same-sire inseminated in two fixed-time artificial insemination (FTAI) events. Pregnancy was checked at 21 d post insemination, and fetal sexing was assessed around 60 d post insemination. At 250 d of gestation, cows were balanced across treatments according to fetal sex (male or female) and then were randomly assigned to one of the three treatments: control or no Vitamin A injection (CON), Vitamin A injection in pregnant cows (VAcow), and Vitamin A injection in newborn calves (VAcalf). Treatments applications were designed as follows: single Vitamin A injection in pregnant cows at 250 d of gestation (VAcow: 2,000,000 IU of Vitamin A); two Vitamin A injections in newborn calves (VAcalf: 200,000 IU of Vitamin A each dose), one at birth and one at 60 d of age. Vitamin A injections were performed intramuscularly at the rump and the substance form used was Retinyl palmitate/Vitamin A palmitate (Monovin A, Bravet, RJ, Brazil). The dosage of Vitamin A injection in newborn calves was chosen based on previous research by Maciel et al. (2022) and Harris et al. (2018). Given the lack of available data regarding Vitamin A injection in cows, the dosage of Vitamin A in pregnant cows was based on the dosage used in VAcalf treatment in proportion to the cow`s average metabolic body weight (BW^0.75^).

At birth, calves were individually identified, had their navels treated with iodine solution (10% concentration), and calves from VAcalf received the first Vitamin A injection (200,000 IU of Vitamin A). Later, at 60d of age, all calves were weighed and calves from VAcalf received their second VA injection (200,000 IU of Vitamin A). At 90 d of age, calves started to receive creep-feeding supplementation (total digestible nutrients = 78.8%; crude protein = 22.0%) fed *ad libitum* until weaning. From gestation until weaning, cow-calf pairs from all treatments were housed in a common paddock with *Urochloa decumbens* in a stocking rate of ~ 1 cow-calf/ha.

Both FTAI groups were simultaneously weaned. In this sense, calves from FTAI group 1 were weaned averaging 311 d of age, and calves from FTAI group 2 averaging 267 d of age. At weaning, all cows and calves were weighed, and ultrasonography was performed in calves at the 12th rib and rump to assess muscle growth development parameters. Ultrasonography images were collected using Aloka 500-V (Corometrics Medical Systems, Wallingford, CT) with a 3.5-MHz, 17.2 cm linear array transducer. Images were further processed using ImageJ software (National Institutes of Health, Bethesda, Maryland, USA). Muscularity index was calculated as the ribeye area (REA) in cm^2^ expressed in units of 100 kg of BW. Longissimus muscle ratio was calculated as the ratio of longissimus muscle width and depth. Still at weaning, skeletal muscle was biopsied at the 12th rib in the *longissimus thoracis* muscle. To conduct the biopsy, area was shaved and a local anesthetic (lidocaine hydrochloride HCl, 20 mg/mL, 6 mL) was injected subcutaneously. The site was disinfected with Betadine, and a 1-cm incision was made with a scalpel. A sterile Bergström muscle biopsy cannula (Eskilds Tuna, Sweden), 5 mm diameter, was used to collect 1 g of muscle tissue, which was immediately placed in liquid nitrogen for transportation and later stored at -80 °C. Incision was then rinsed with sterile saline and water, sealed with veterinary tissue glue, and sprayed with an antibiotic. Calves were examined for 72 h after the biopsy and treated with antibiotics in case of infection (20 mg of oxytetracycline/kg BW).

Unfortunately, unforeseen predator attacks, along with accidents related to natural locomotion and social hierarchy conflicts, resulted in the loss of a few experimental units, which were excluded from the final data analysis. Individual data collected before these incidents were retained, as losses were random and not associated with treatments effects. Therefore, no data imputation was applied. In summary, from the initially 51 cow-calf pair (17 pairs per treatment), the dataset ended up with 40 cows (28 primiparous and 12 multiparous) and 40 calves (16 calves in CON, 8 males and 8 females; 17 calves in VAcalf, 9 males and 8 females; 7 calves in VAcow, 3 males and 4 females).

### Real-time quantitative polymerase chain reaction (RT-qPCR)

Total RNA was extracted from approximately 170 mg of muscle tissue using the SV Total RNA Isolation System (Promega, Madison, WI, USA), with modifications to the manufacturer’s protocol. Briefly, 350 µL of lysate was used, and 100 µL of QIAzol (Qiagen, Valencia, CA, USA) was added before a 3-minute incubation at 70 °C. RNA concentration was measured using a Nano spectrophotometer (DeNovix DS-11, Wilmington, DE, USA), and RNA integrity was assessed by electrophoresis on agarose gel. Complementary DNA (cDNA) synthesis was performed using the High-Capacity cDNA Reverse Transcription Kit (Applied Biosystems, Foster City, CA, USA).

Details of the primers used in the reactions are provided in Table 1. Primer sequences were designed based on reference sequences available in the NCBI database using the Primer3Plus web interface and further evaluated with Oligo Analyzer 3.1 and Premier Biosoft tools. Primers were synthesized by Invitrogen (Carlsbad, CA, USA).

**Table 1:** Primers sequences (5’ – 3’) used in the RT-qPCR analysis

Gene symbol	NCBI Accession Number	Primer sequence
*ACTB*	NM_173979.3	F: GTCCACCTTCCAGCAGATGT
		R: CAGTCCGCCTAGAAGCATTT
*CEBPA*	NM_176784.2	F: GGCAACGACTTTGACTACCC
		R: GGTCATTGTCACTGGTCAGC
*DLK1*	NM_174037.2	F: GGATTCTGCGACGATGACAG
		R: TGTGGTTGTAGCGCAGATTG
*FABP4*	NM_174314.2	F: GCTGCACTTCTTTCTCACCT
		R: TGGACAACGTATCCAGCAGA
*GAPDH*	NM_001034034.2	F: CACAGTCAAGGCAGAGAACG
		R: ATTCTCAGTGTGGCGGAGAT
*GHR*	NM_176608.1	F: CCAGCTTTCCTTGTCAGAGC
		R: GAAGTTAGCTTGGCAGGGTG
*IGF1R*	NM_001244612.1	F: GACTCCTGTTTTTCTCCGCC
		R: CAGTTCCGAAGCGATGTTGT
*MTOR*	XM_002694043.7	F: GCACATGCAGCACTTTGTTC
		R: GGATTTTGTTGGCTGCGTTG
*MYF5*	NM_174116.1	F: AGCGTCTACTGTCCTGATGTA
		R: GTTGGTGATCCGATCCACTATG
*MYF6*	NM_181811.2	F: TACCCTGCAGCCCTTAGAAG
		R: TACAAGCCCAAAGCCGAAAG
*MYH1*	NM_174117.1	F: CCACTTTGTACGCTGCATCA
		R: GTGGCGTGTTTCTCCTTCTC
*MYH2*	NM_001166227.1	F: TCGCAACGCAGAAGAGAAAG
		F: AGCATCAGGACACGATCACT
*MYH7*	NM_174727.1	F: CAGAAGAACGCTGTGACCAG
		R: TCTTGTTCTCGCGCTTGAAG
*MYOD1*	NM_001040478.2	F: CCTGAGCAAAGTCAACGAGG
		R: GTAAATCGGGTTGGGGTTCG
*MYOG*	NM_001111325.1	F: CACAGATGCCACCACTTCTG
		R: TTCAGCACAGAGACCTTGGT
*PAX7*	XM_027522151.1	F: GACCCTCCAGTTTCCTCATTT
		R: CCAGTTATGAAACCCTCCTCTG
*PDGFRA*	XM_024993021.1	F: CGAGATGGGAGTTTCCAAGAG
		R: GACAGGTTGAGACCGACTTAAT
*PPARD*	XM_024983411.1	F: CAGCTACACAGGGCTTCTT
		R: CACTTGTTGCGGTTCTTCTTC
*PPARG*	NM_181024.2	F: GACATCAAGCCCTTCACCAC
		G: GGGGACTGATGTGCTTGAAC
*RARA*	NM_001014942.4	F: TCTGCCTCATCTGTGGAGAC
		R: CTGGCATTTGCTGGTGATGA
*RARB*	XM_059882389.1	F: TCTGTGAGAATCCTGGGAGC
		R: CCAGCAGTGGTTCTTGTAGC
*RARG*	NM_001130756.1	F: GCAAGTACACCACGAACTCC
		R: ATGAGCATCCTGGGGAACAT
*RXRA*	NM_001304343.1	F: CGAGGTCCTCTGTTTGCAAG
		R: CTGCTTCACTCTGCTGACAC
*RXRB*	NM_001083640.1	F: CTGTGACCAACATCTGCCAG
		R: CTCCATGAGGAAGGTGTCGA
*RXRG*	NM_001075408.1	F: CAGGAAAGCACTACGGTGTG
		R: GAAACCGAGCGATGGGAAAA
*VEGFA*	NM_174216.2	F: ACTTGAGTTGGGAGGAGAATG
		R: GCTGCCGTAAGAGGGATAAA
*WNT1*	NM_001114191.1	F: AGAGTCTGCAGCTGGTACTC
		R: CTGTACGTGCAGAAGTTGGG
*ZNF423*	NM_001101893.1	F: CCGTTCAAGTGCACCTACTG
		R: GGACGAAGACTGTGAAGCAC

Actin beta (*ACTB*), CCAAT Enhancer binding protein alpha (*CEBPA*), Delta like non-canonical notch ligand 1 (*DLK1*), Fatty acid binding protein 4 (*FABP4*), Glyceraldehyde-3-phosphate dehydrogenase (*GAPDH*), Growth hormone receptor (*GHR*), Insulin like growth factor 1 receptor (*IGF1R*), Mechanistic target of rapamycin kinase (*MTOR*), Myogenic factor 5 (*MYF5*), Myogenic factor 6 (*MYF6*), Myosin heavy chain 1 (*MYH1*), Myosin heavy chain 2 (*MYH2*), Myosin heavy chain 7 (*MYH7*), Myogenic differentiation 1 (*MYOD1*), Myogenin (*MYOG*), Paired box 7 (*PAX7*), Platelet derived growth factor receptor alpha (*PDGFRA*), Peroxisome proliferator activated receptor delta (*PPARD*), Peroxisome Proliferator Activated Receptor gamma (*PPARG*), Retinoic acid receptor alpha (*RARA*), Retinoic Acid Receptor beta (*RARB*), Retinoic Acid Receptor gamma (*RARG*), Retinoid x receptor alpha (*RXRA*), Retinoid X Receptor beta (*RXRB*), Retinoid X Receptor gamma (*RXRG*), Vascular endothelial growth factor A (*VEGFA*), Wnt family member 1 (*WNT1*), Zinc finger protein 423 (*ZNF423*)

Quantitative PCR was carried out using the SYBR Green detection system (Applied Biosystems, USA) on an Eppendorf Realplex2 thermocycler (Eppendorf, Hamburg, Germany). All RT–qPCR reactions were performed using cDNA from 40 biological replicates, with three technical replicates per sample. Gene expression results are expressed relative to the average of Actin beta (*ACTB*) and Glyceraldehyde-3-phosphate dehydrogenase (*GAPDH*) and were calculated using the Delta Delta Ct method (Livak and Schmittgen 2001).

### Statistical analysis

Data were analyzed using the MIXED procedure of SAS software (SAS Inst. Inc., Cary, NC, USA). To assess data normality, the Shapiro-Wilk test was performed. When the data were not normally distributed, they were transformed using the RANK procedure. Cow parity (primiparous and multiparous) and FTAI event (group 1 and group 2) were considered blocks. Cow’s initial BW was used as a covariate adjustment. The cow-calf pair was considered the experimental unit. Cow parity, FTAI group, and treatment were considered as fixed effects, while the cow-calf pair as a random effect. To account for the disparity in the number of experimental units among treatments, the Satterthwaite approximation was applied to adjust the denominator degrees of freedom. In addition, a post-hoc power analysis was performed using the POWER procedure of SAS to ensure that the results were interpreted correctly despite the imbalance in experimental units within the dataset. Statistical differences were declared when *P* ≤ 0.05, and trends were discussed when 0.05 < *P* ≤ 0.10.

## Results

**Fig. 1:**
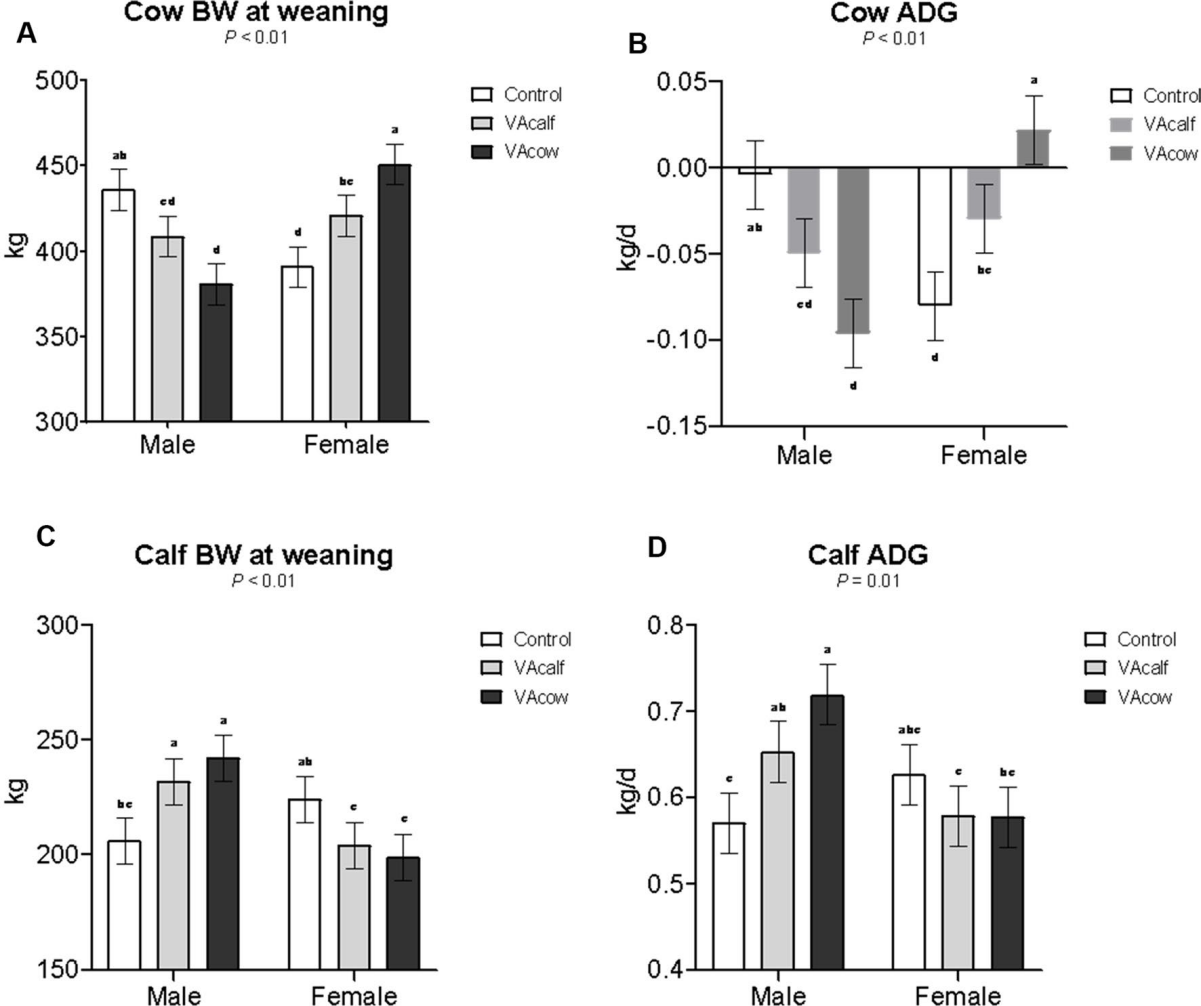
Interactions between treatment × sex on cow and calf performance during the cow-calf phase. ^a−c^ Means within a row sharing a common superscript do not differ significantly. Control = control treatment with no Vitamin A injection in cows or calves; VAcalf = VA injection in newborn calves at birth and 60 d of age; VAcow = VA injection in pregnant cows at 250 d of gestation

No vitamin A or sex effects were observed (*P* > 0.18) for cow initial body weight (BW) or calf BW at 60 days of age (Table 2). Cows in the VAcow group nursing female calves had greater BW at weaning and ADG compared with most other groups, except for Control cows nursing male calves, which they did not differ (Fig. 1). Calf BW at weaning was higher in VAcalf and VAcow male calves compared to Control male and female calves in both VA-treated groups. Additionally, male calves from the VAcow group showed greater ADG than Control male and female calves from both VA-treated groups.

**Table 2:** Effects of maternal or neonatal Vitamin A injection on cow and calf performance during the cow-calf phase

Item	Treatments			SEM^‡^	Sex		SEM^‡^	*P*-value		
	Control	VAcalf	VAcow		Male	Female		VA^¥^	Sex	VA×S^†^
Cow initial BW, kg	423	443	466	16.0	445	443	13.7	0.18	0.93	0.94
Cow BW at weaning, kg	414	415	416	8.85	409	421	7.45	0.97	0.19	< 0.01
Cow ADG, kg/d	-0.042	-0.040	-0.037	0.0150	-0.050	-0.029	0.0126	0.97	0.20	< 0.01
Calf BW at 60 d, kg	63.4	61.6	55.9	4.23	62.1	58.5	3.55	0.51	0.42	0.36
Calf BW at weaning, kg	215	218	221	7.41	227	209	6.24	0.88	0.03	< 0.01
Calf ADG, kg/d	0.598	0.616	0.648	0.0263	0.648	0.594	0.0221	0.46	0.06	0.01

^a, b, c, d^ Means within a row sharing a common superscript do not differ significantly

^‡^SEM = standard error of the means

^¥^VA = *P*-value for vitamin A effects

^†^VA×S = vitamin A × sex interaction

Control = control treatment with no Vitamin A injection in cows or calves; VAcalf = VA injection in newborn calves at birth and 60 d of age; VAcow = VA injection in pregnant cows at 250 d of gestation

**Fig. 2:**
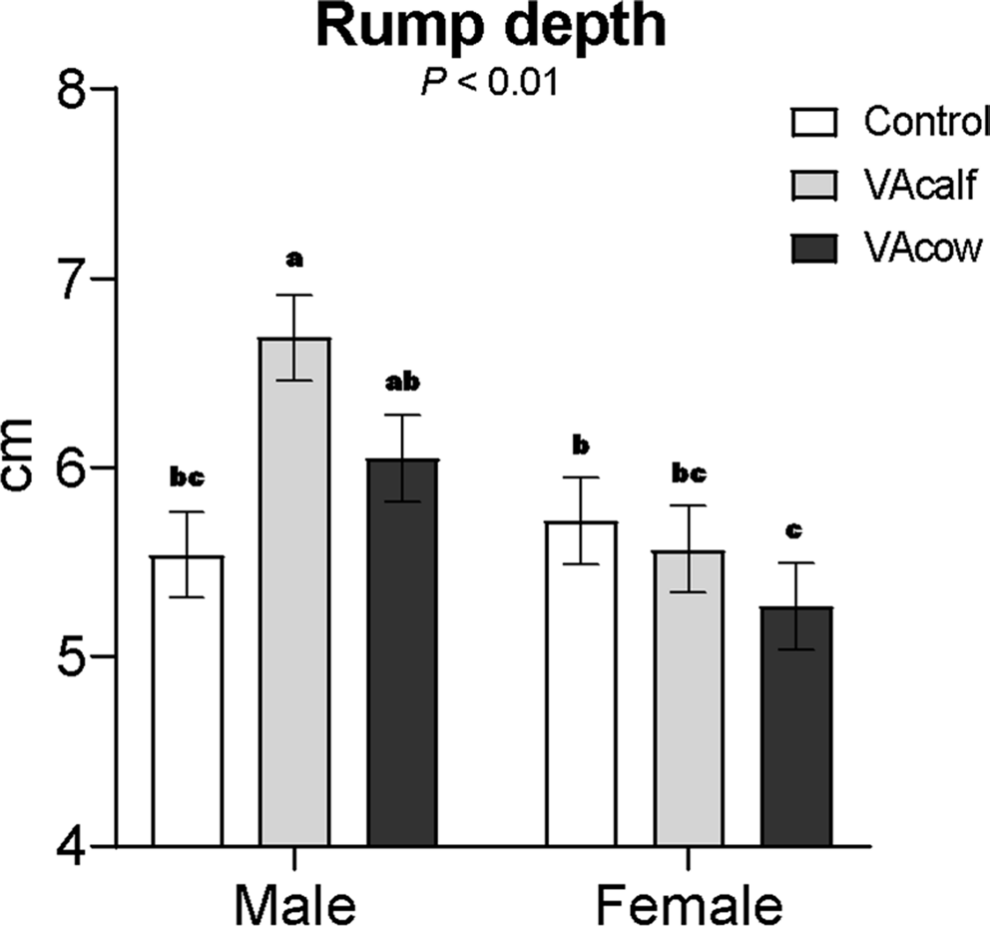
Interaction between vitamin A × sex on calf rump depth. ^a−c^ Means within a row sharing a common superscript do not differ significantly. Control = control treatment with no Vitamin A injection in cows or calves; VAcalf = VA injection in newborn calves at birth and 60 d of age; VAcow = VA injection in pregnant cows at 250 d of gestation

Vitamin A injection in the dams or calves did not affect body measurements at weaning (Table 3). However, the longissimus muscle area (LMA) was greater in male calves compared to female calves. A significant vitamin A × sex interaction was detected for rump depth, with VAcalf male calves exhibiting greater rump depth than Control male calves and female calves from all treatments (Fig. 2).

**Table 3:** Effects of maternal or neonatal Vitamin A injection on calf body measurements at weaning

Item	Treatments			SEM^‡^	Sex		SEM^‡^	*P*-value		
	Control	VAcalf	VAcow		Male	Female		VA^¥^	Sex	VA×S^†^
Backfat thickness, mm	0.66	0.70	0.63	0.062	0.66	0.66	0.052	0.43	0.19	0.82
Longissimus muscle area, cm^2^	36.4	35.6	32.1	2.04	37.3^a^	32.1^b^	1.70	0.37	0.02	0.20
Muscularity index, cm^2^/100 kg BW	0.167	0.162	0.150	0.0067	0.160	0.159	0.0056	0.25	0.81	0.42
Longissimus muscle width, cm	11.4	11.2	11.6	0.45	11.7	11.1	0.37	0.79	0.17	0.31
Longissimus muscle depth, cm	4.58	4.91	4.58	0.18	4.82	4.56	0.149	0.22	0.17	0.71
Longissimus muscle ratio	2.52	2.29	2.54	0.116	2.46	2.44	0.097	0.19	0.88	0.67
Rump fat thickness, mm	0.66	0.73	0.72	0.091	0.66	0.75	0.076	0.99	0.98	0.79
Rump depth, cm	5.63	6.13	5.66	0.169	6.09	5.52	0.141	0.09	< 0.01	< 0.01

^a, b, c^ Means within a row sharing a common superscript do not differ significantly

^‡^SEM = standard error of the means

^¥^VA = *P*-value for vitamin A effects

^†^VA×S = vitamin A × sex interaction

Control = control treatment with no Vitamin A injection in cows or calves; VAcalf = VA injection in newborn calves at birth and 60 d of age; VAcow = VA injection in pregnant cows at 250 d of gestation

**Fig. 3:**
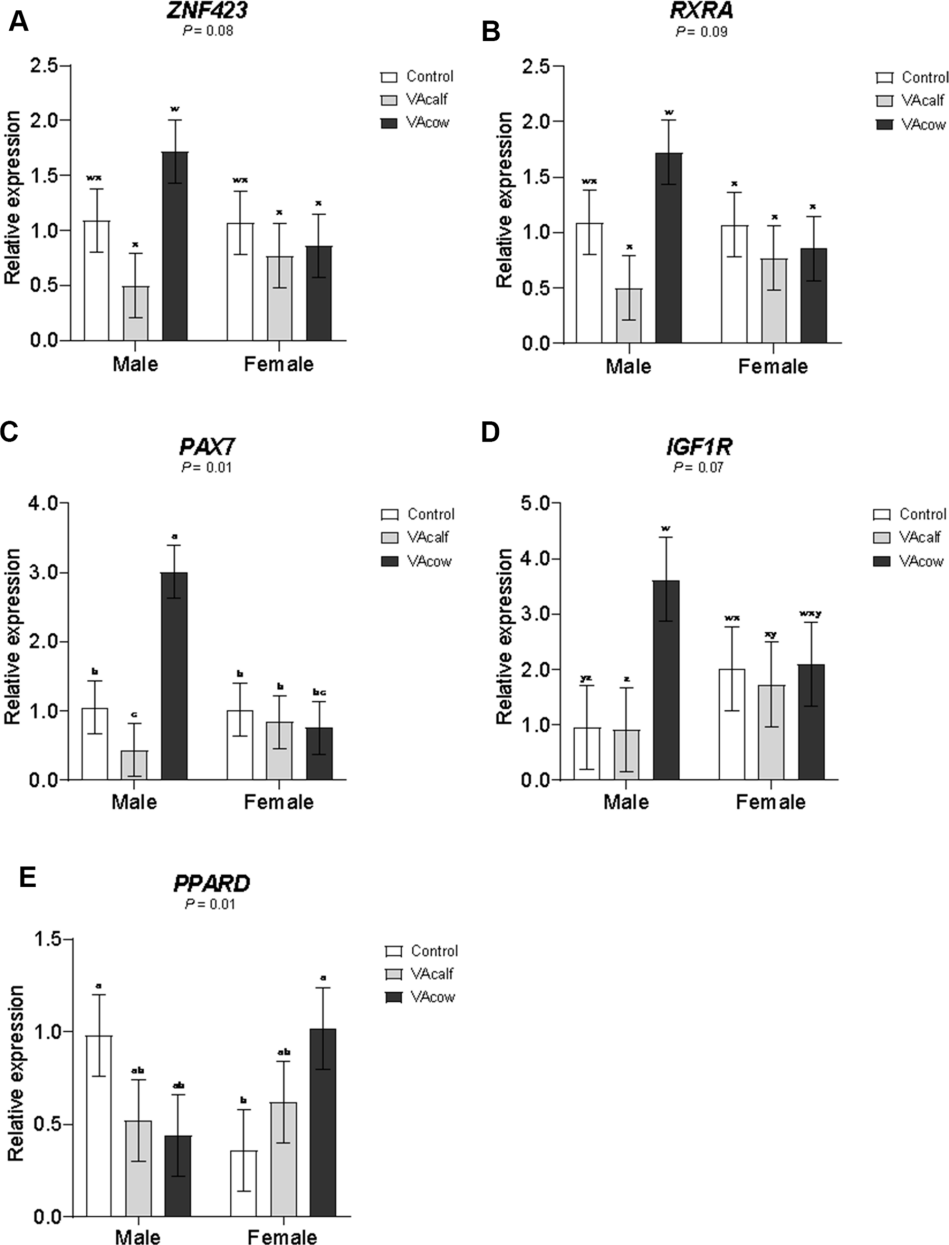
Interactions between vitamin A × sex on longissimus thoracis muscle gene expression at weaning. ^**a**−**c**^ Means within a row sharing a common superscript do not differ significantly. ^*w−z*^ Means within a row sharing a common superscript tend to not differ. Control = control treatment with no Vitamin A injection in cows or calves; VAcalf = VA injection in newborn calves at birth and 60 d of age; VAcow = VA injection in pregnant cows at 250 d of gestation

Skeletal muscle mRNA expression at weaning is presented in Table 4. Vitamin A injection affected some important genes` expression, which animals in the VAcow and Control groups showed greater expression of *RARA*, *MYF5*, *CEBPA*, and *DLK1* compared with VAcalf animals. In addition, female calves exhibited higher expression of *MYH1* and tended (*P* = 0.09) to have greater expression of *MYF6* than male calves. Expression of *ZNF423* tended to be greater (*P* = 0.08) in VAcow male calves than in VAcalf male calves and in VA-treated female calves (Fig. 3). Similarly, *RXRA* expression tended to be higher (*P* = 0.09) in VAcow male calves compared to VAcalf male calves and female calves from all groups. Additionally, *PAX7* expression was greater in VAcow male calves than in calves from other treatments. Expression of *IGFR1* tended to be higher (*P* = 0.07) in VAcow male calves compared to Control and VAcalf male calves, as well as VAcalf female calves. Finally, *PPARD* expression was greater in Control male calves and VAcow female calves compared to Control female calves.

**Table 4:** Effects of maternal or neonatal Vitamin A injection on longissimus thoracis muscle gene expression at weaning

Gene	Treatments			SEM^‡^	Sex		SEM^‡^	*P*-value		
	Control	VAcalf	VAcow		Male	Female		VA^¥^	Sex	VA×S^†^
*Retinoic acid signaling and Vitamin A response*										
*ZNF423*	1.08	0.64	1.29	0.217	0.90	1.10	0.206	0.15	0.35	0.08
*RARA*	1.11^a^	0.61^b^	1.27^a^	0.243	1.05	0.95	0.202	0.04	0.89	0.14
*RARB*	1.14	1.01	1.00	0.357	1.03	1.07	0.300	0.93	0.98	0.17
*RARG*	0.77	1.05	1.26	0.281	0.99	1.06	0.231	0.50	0.98	0.48
*RXRA*	0.90	0.50	0.85	0.181	0.92	0.58	0.149	0.33	0.21	0.09
*RXRB*	0.85	0.52	1.13	0.316	1.03	0.64	0.262	0.49	0.66	0.50
*RXRG*	0.78	0.53	0.49	0.126	0.72	0.48	0.105	0.36	0.11	0.22
*Muscle cell determination and satellite cell proliferation*										
*MYF5*	1.05^a^	0.62^b^	1.41^a^	0.263	1.05	1.00	0.219	0.03	0.70	0.28
*PAX7*	1.03	0.64	1.88	0.282	1.50	0.87	0.235	0.05	0.45	0.01
*MYOD1*	1.64	1.66	1.05	0.852	0.99	1.91	0.712	0.90	0.57	0.98
*MYOG*	1.00	0.63	0.53	0.186	0.81	0.63	0.154	0.35	0.45	0.71
*MYF6*	1.63	3.23	0.10	1.064	1.29^x^	2.02^w^	0.885	0.25	0.09	0.93
*Muscle hypertrophy*,* growth*,* and regeneration*										
*MTOR*	0.87	1.37	1.46	0.325	1.06	1.41	0.272	0.64	0.46	0.73
*IGF1R*	1.49	1.32	2.86	0.566	1.83	1.95	0.465	0.04	0.28	0.07
*GHR*	0.98	1.03	1.13	0.358	1.00	1.09	0.299	0.66	0.85	0.40
*Angiogenesis and vascular support*										
*VEGFA*	1.22	1.40	1.63	0.273	1.37	1.46	0.225	0.72	0.81	0.18
*PDGFRA*	0.92	0.68	0.66	0.154	0.82	0.69	0.128	0.66	0.45	0.48
*Lipid metabolism and adipogenesis*										
*FABP4*	0.81	0.91	0.39	0.338	0.91	0.49	0.281	0.61	0.25	0.96
*PPARG*	1.05	1.65	1.25	0.373	1.46	1.17	0.312	0.40	0.61	0.25
*PPARD*	0.67	0.57	0.73	0.165	0.65	0.67	0.137	0.92	0.81	0.01
*Skeletal muscle fiber maturation*										
*MYH1*	1.04	0.89	1.20	0.251	0.76^b^	1.33^a^	0.209	0.59	0.02	0.13
*MYH2*	0.91	1.05	0.97	0.260	1.02	0.94	0.216	0.82	0.59	0.58
*MYH7*	0.59	1.12	0.35	0.289	0.99	0.38	0.240	0.42	0.29	0.33
*Other key regulators of mesenchymal cell development*										
*WNT*	1.46	1.01	1.15	0.273	1.22	1.19	0.224	0.80	0.85	0.20
*CEBPA*	0.81^a^	0.35^b^	1.09^a^	0.286	0.97	0.53	0.239	0.05	0.43	0.14
*DLK1*	0.92^a^	0.53^b^	1.20^a^	0.237	0.97	0.79	0.199	0.04	0.82	0.14

^a, b, c^ Means within a row sharing a common superscript do not differ significantly

^w, x^ Means within a row sharing a common superscript tend to not differ

^‡^SEM = standard error of the means

^¥^VA = *P*-value for vitamin A effects

^†^VA×S = vitamin A × sex interaction

Control = control treatment with no Vitamin A injection in cows or calves; VAcalf = VA injection in newborn calves at birth and 60 d of age; VAcow = VA injection in pregnant cows at 250 d of gestation

## Discussion

Vitamin A has emerged as a potential regulator of fetal and early postnatal development, particularly in muscle and adipose tissues during critical windows of development. In this study, we evaluated the effects of Vitamin A injection in pregnant cows or newborn calves on cow-calf performance, muscle development assessed via ultrasound, and skeletal muscle gene expression at weaning. Although no main effects of vitamin A or sex were observed for calf body weight at 60 days of age, significant treatment × sex interactions were detected for some important performance traits.

Cows in the VAcow group nursing female calves exhibited greater ADG and BW at weaning, which may reflect an improved maternal metabolic status and energy balance, as well as different nutritional demands from the offspring. This difference in demand is supported by Vitamin A-treated female calves presenting lower BW at weaning compared to Vitamin A-treated male calves. The sex-dependent Vitamin A response could be related to skeletal muscle metabolism and tissue development priorities, possibly involving epigenetic mechanisms. During folliculogenesis, in the fetal and early neonatal phases, vast epigenetic remodeling occurs, such as the remethylation of the genome (Walker and Ho 2012). Given that both referred phases overlap our treatment applications, we speculated that Vitamin A-treated female calves may have prioritized reproductive tract maturation over skeletal muscle growth and development during early life due to different patterns in DNA methylation. The fact that *MYH1* expression at weaning was greater in females compared to male calves at weaning may further indicate a possible delayed muscle fiber maturation in females. In addition, Bozack et al. (2021) evaluating perinatal stress and other environmental exposures in mother-child pairs demonstrated higher methylation levels among females in cord blood and artery samples, and higher methylation levels among males in placenta samples. In another study conducted with mice, significant sex differences were observed in DNA methylation and gene expression profiles (Legault et al. 2023). The authors reported that these changes occur not only on the X chromosome but also on autosomes. These findings provide evidence that fetal sex markedly influences DNA methylation, resulting in sex-specific epigenetic patterns across tissues and developmental stages. Nonetheless, further research is required to explore sex-related differences in epigenetic mechanisms, particularly in response to vitamin A supplementation in bovines.

Interestingly, male calves from both Vitamin A-treated groups showed greater BW at weaning than their Control counterparts and Vitamin A-treated female calves. Previous research has also pointed to improved performance in early life following Vitamin A supplementation (Harris et al. 2018; Peng et al. 2020), although such effects have not been universally observed across all studies (Jo et al. 2020; Maciel et al. 2022). This suggests that the response to Vitamin A supplementation on postnatal growth may depend on both sex and dosage, particularly when administered to the dam during gestation. These results reinforce the concept of fetal programming, where maternal nutritional interventions can produce sex-specific phenotypic responses (Alsiraj et al. 2025; Campbell et al. 2022; McCoski et al. 2021). The fact that only male calves responded positively to maternal Vitamin A injection suggests a greater developmental plasticity in males during the prenatal period. Notably, this is the first study to examine the effects of Vitamin A injection in Nellore × Angus cattle, and potential breed-specific physiological characteristics may have influenced the observed performance differences.

The expression of genes observed at weaning suggests that maternal Vitamin A administration may prime the offspring’s skeletal muscle tissue for improved growth. *PAX7* expression, a critical marker of muscle satellite cells, was significantly higher in VAcow male calves, and *IGF1R* expression, a gene associated with anabolic signaling and muscle growth, also tended to be greater in VAcow male calves. The upregulation in *PAX7* and *IGF1R* expression in VAcow male calves may underlie the improved ADG and BW at weaning, reflecting hormonal signaling, enhanced satellite cell activity, and myogenic and hypertrophy capacity (von Maltzahn et al. 2012). Histological evaluation of muscle cross-sections of these animals, collected at slaughter after 207 days of feedlot, revealed that VAcow male calves had a greater number of muscle fibers per field, whereas VAcalf male calves exhibited the lowest fiber counts among treatments (data not shown). This observation supports the phenotypic findings and suggests that VA injection in pregnant cows at 250 days of gestation may have triggered a late muscle fiber hyperplasia stimulus, potentially contributing to enhanced muscle development in their offspring.

Ultrasound measurements revealed that male calves had greater LMA than female calves, consistent with known sex differences in muscle growth potential. A Vitamin A × sex interaction was detected for rump depth, where VAcalf male calves had greater rump depth than Control males and all female calves. The increased rump depth in VAcalf males aligns with the greater BW at weaning in this group and may also reflect localized effects of Vitamin A on early developed muscle groups, possibly driven by regional variation in gene expression or retinoic acid sensitivity.

The tendency for increased expression of *ZNF423* and *RXRA* in VAcow male calves supports a potential role of maternal Vitamin A in preadipocyte commitment and nuclear receptor activity, respectively. The key nuclear receptor *RXRA* mediates retinoic acid action on gene transcription (Al Tanoury et al. 2013), while *ZNF423* plays a role in preadipocyte determination (Gupta et al. 2010), suggesting that maternal Vitamin A may influence both myogenic and adipogenic lineages. The present study followed calves until weaning, so long-term implications of these early-life responses for intramuscular fat deposition or overall carcass quality remain unclear.

In addition, calves in the VAcow and Control groups had higher expressions of *RARA*, *MYF5*, *CEBPA*, and *DLK1* compared to VAcalf calves. These genes play key roles in retinoic acid signaling (*RARA*), muscle differentiation (*MYF5*), adipogenic commitment (*CEBPA*), and preadipocyte regulation (*DLK1*), indicating that the timing and dosage of VA exposure differentially shape transcriptional responses in muscle tissue. Interestingly, in our study, the VAcalf group, which received two neonatal doses (200,000 IU each), exhibited lower expression of *CEBPA* and *DLK1*, two key regulators of adipogenesis. This downregulation may reflect both the supraphysiological Vitamin A dose and the developmental stage at which the muscle samples were collected, since at the time of biopsy the adipogenic potential was likely limited, given that the recruitment and differentiation of adipogenic precursor cells in cattle are reported to continue up to approximately 250 days of age (Du et al. 2010). This contrasts with previous findings where early-life Vitamin A administration at birth (Maciel et al. 2022; dosage 300,000 IU) or at birth and at 1 month of age (Harris et al. 2018; dosage 150,000 IU) increased adipogenic potential during early life. In the study by Harris et al. (2018), a higher dosage (300,000 IU) produced outcomes similar to the control group in terms of adipogenic potential and intramuscular fat content later in life. Moreover, early-life expression of *ZFP423* and *PPARG* was substantially greater when calves received 150,000 IU of vitamin A compared to the higher dose, suggesting that excessive Vitamin A exposure may inhibit adipogenic gene expression and reduce marbling potential. Collectively, these results suggest that the timing, frequency, and dosage of Vitamin A exposure interact with developmental stage–specific windows of adipogenic sensitivity, influencing the balance between preadipocyte commitment and differentiation. Further studies are necessary to determine the optimal Vitamin A dose and administration schedule to promote favorable adipogenic outcomes in neonatal calves.

Altogether, our findings emphasize the biological relevance of the developmental window during which Vitamin A is administered. From a practical standpoint, these findings highlight the potential for targeted maternal supplementation strategies to enhance progeny performance, particularly in male calves’ production. Administering Vitamin A to pregnant cows appears to elicit a more pronounced effect on calf growth and muscle gene expression, particularly in male offspring. However, the divergent responses observed between sexes suggest that Vitamin A may also differentially influence developmental priorities in females, potentially affecting different tissues and pathways. These sex-dependent responses warrant further investigation, possibly involving epigenetic mechanisms such as DNA methylation or chromatin remodeling, as well as a deeper evaluation of how Vitamin A impacts female offspring physiology beyond the early growth period.

## Conclusion

Vitamin A injection during late pregnancy or early postnatal life enhanced growth performance in Angus × Nellore male calves. In addition, Vitamin A injection during the gestation or neonatal stage had a different effect on the expression of genes involved in adipogenesis and myogenesis, suggesting that prenatal use has a more pronounced effect. Moreover, female calves displayed distinct responses, indicating that Vitamin A may influence developmental priorities differently across sexes. Future research with larger datasets and methylation assays is warranted to elucidate the underlying mechanisms of these sex-specific responses and to define the optimal timing, dose, and frequency of administration, as well as to assess the long-term effects of Vitamin A injection on carcass merit.

## Data Availability

Data from this study are available upon request to the corresponding author.
